# A cross-sectional study of public attitudes towards safer drug use practices in British Columbia, Canada

**DOI:** 10.1186/1747-597X-8-40

**Published:** 2013-12-01

**Authors:** Despina Tzemis, Jennifer Campbell, Margot Kuo, Jane A Buxton

**Affiliations:** 1 Communicable Disease Prevention and Control Services, British Columbia Centre for Disease Control, 655 West 12th Avenue, Vancouver, BC V5Z 4R4, Canada

**Keywords:** Harm reduction, Safer drug use, Public attitude

## Abstract

**Background:**

Harm reduction programs are often vulnerable to political and vocal opposition despite documented evidence of their effectiveness and economic benefit. It is not well understood if opponents to harm reduction represent the general public’s attitudes.

**Objective:**

To understand the attitudes of the people of British Columbia (BC) towards various harm reduction strategies and services, and factors associated with support for harm reduction.

**Methods:**

A random-digit dialing telephone survey assessing attitudes towards various harm reduction strategies was administered to British Columbians in August 2011 (n = 2000). We compared the level of support for general harm reduction by sex, age, education level, and area of residence (Health Authority region) (χ^2^). Multivariate logistic regression was used to assess odds of support for harm reduction.

**Results:**

Overall support for general harm reduction among participants was 76%; needle distribution 72%; needle distribution in one’s local community 65%; and safer inhalation equipment distribution 52%. In the multivariate analysis, those with significantly lower odds of supporting harm reduction were male, older, had equal or less than high school education or completed a certificate/diploma program, and resided in the Fraser Health Authority region. The Health Authority region with a municipality that has introduced a bylaw prohibiting the implementation of harm reduction services was found to have 69% support for harm reduction. Another Health Authority region with a municipality that closed a long-standing needle distribution site was found to have over 78% support.

**Conclusion:**

In contrast to some local policies, our results show the British Columbians surveyed in our study support harm reduction. It is unclear whether policy makers are swayed by a vocal minority or block harm reduction activities for other reasons. Tailoring messages towards segments of the public less likely to support harm reduction, as well civic policy-makers and the media, may help to reduce stigma and gain support for harm reduction services designed to protect and improve the health of the individual and the public.

## Background

Harm reduction aims to minimize death, disease, and injury from high-risk behaviour by promoting safer drug use practices among people who use drugs who have not accepted, or are not currently able to accept, a treatment goal of abstinence [1,2]. It involves a range of strategies and services including needle distribution programs, safer consumption facilities, substitution therapy programs, and referral to counseling and drug treatment programs [2,3]. Harm reduction consists of non-judgemental approaches to delivering health services and aims to treat people who use drugs with respect, dignity, and compassion [2].

British Columbia (BC), Canada is seen by many as a leader in harm reduction and hosts several innovative examples of successful harm reduction efforts [4]. The BC Centre for Disease Control distributes millions of provincially funded sterile needles/syringes across the province annually, contributing to the decline of HIV and hepatitis C incidence in BC [5,6]. In the Vancouver Coastal Health region of BC there is the first officially sanctioned supervised injection facility in North America [7] and a clinical trial for heroin-assisted therapy [8]. Recently, harm reduction efforts in BC have expanded to reduce the risks associated with smoking crack cocaine by providing safer inhalation equipment such as plastic mouth pieces and glass stems (also known as ‘crack pipes’) [9].

However, it should be noted that several areas of BC face challenges with implementing harm reduction programs. For example, in 2007 Abbotsford city council implemented a bylaw forbidding harm reduction services [10] and in 2008 local opposition in Victoria resulted in the closure of a long-standing needle distribution site [11,12]. Despite documented evidence of health benefits and economic benefit, harm reduction programs may be limited because of perceived negative public opinion and policy makers’ fear of sending the ‘wrong message’ [3,6,13,14].

Unfortunately, political interference in harm reduction initiatives has been documented around the world and is not unique to BC [3]. Researchers in the United States (US) agree that perceived negative public opinion plays a role in the disjunction between science and policy and contributes to the low uptake of harm reduction services in the US [15]. It is noted that vocal opposition is often displayed at the local level as the NIMBY ‘not-in-my-back-yard’ attitude following the commencement of new harm reduction services [15-17].

The literature previously examining public attitudes towards harm reduction suggests these fears of negative public opinion may be unwarranted. In a systematic review of public perceptions towards harm reduction programs, findings show that surveys from Canada, the UK, and Australia predominantly demonstrate a clear majority in support of harm reduction programs [3]. Yet, these public survey findings seem to be ignored by some policy-makers and media sources which often negatively represent safer drug use practices on ideological grounds, disregarding their public health benefits [18,19].

Public support is critical for the feasibility and sustainability of harm reduction services as public support influences political will [3,15,17]. Both Vernick et al. [15] and Thein et al. [17] suggest investigating public attitudes towards safer drug use practices is essential in order to determine level of public support, measure public support over time, and identify factors that may increase public support. At this time there is a lack of systematically obtained data across the province so it is unclear if harm reduction policies that limit service provision in BC are truly reflective of popular opinion.

This study was designed to provide an understanding of public attitudes towards safer drug use practices in BC in four main areas: general harm reduction; needle distribution services; needle distribution services in one’s community; and distribution of safer inhalation equipment, a new initiative in BC. This study sought to understand if regions of BC with policies against harm reduction services had concomitant lower public support. The findings will help health and service providers identify and target appropriate messaging towards those in the population who may be less informed on the importance and implications of safer drug use practices. Focusing efforts on improving public acceptance of harm reduction programming, alongside documented evidence of its effectiveness, may help influence policy-makers to support and implement harm reduction programs in BC. Furthermore, the findings will quantify a baseline level of support across the province. Our research may also raise international awareness of how the public, a harm reduction stakeholder, may influence harm reduction policy.

## Methods

### Study design

In August 2011, a random-digit dialing telephone survey was administered to BC households. A research company specializing in computer-assisted telephone interviewing administered the survey and collated the data. The company used a comprehensive list of BC household numbers, including telephone numbers predicted to come into existence after the date on which the current list was established. When conducting the study, households that did not answer the telephone remained in the pool of potential numbers to phone, while those who answered were excluded from future calls. To meet ethical requirements, and retain randomization, participant selection was based on the person in the household aged 19 years of age or older who had the next birthday. Respondents who gave verbal consent participated in an interview which took approximately 15 minutes. Ethical approval for the study was obtained from the University of British Columbia Behavioral Research Ethics Board.

### Study instrument

The survey collected socio-demographic information and measured attitudes towards various harm reduction strategies and services. A short description of harm reduction strategies and services was given prior to asking respondents to provide their opinion. These descriptions were developed by the study team. The level of support was gauged on a 5 item Likert scale: strongly support, somewhat support, neutral, somewhat oppose, or strongly oppose. Survey questions for the four main outcomes of interest are as follows:

• (General harm reduction): Harm reduction strategies are public health programs that reduce the harms related to drug use. Supporters generally think these programs can significantly reduce death and the transmission of disease among people who use drugs, and that these programs can bring them into contact with public health or other services to act as a ‘bridge to recovery’. Opponents argue that harm reduction programs encourage drug use and should not be used. I would like to know your opinion on the subject. Do you support or oppose harm reduction strategies for people who use drugs?”

• (Needle distribution services): Needle distribution programs provide clean needles to people who use drugs and encourage safe disposal of used needles in order to prevent needle sharing, which can spread infectious diseases. Do you support or oppose needle distribution programs?

• (Needle distribution in one’s community): Do you support or oppose the idea that there should be needle distribution services in your community to help people with drug addictions?

• (Safer inhalation equipment distribution): Recently there has been a change in drug use practices. An increasing number of drug users are smoking or inhaling drugs and fewer may be injecting. Harms related to inhaling drugs include transmission of infections such as HIV and hepatitis C from sharing drug equipment. Some communities provide small glass stems, also known as crack pipes, and plastic tubing for mouthpieces, to reduce the harms related to drug inhalation. Do you support or oppose the distribution of safer inhalation supplies such as glass stems and plastic tubing?

### Outcome variables

The main outcome variables of interest for this study were the levels of support for harm reduction, needle distribution, needle distribution in one’s own community, and safer inhalation equipment distribution. Level of support was dichotomized as either: support (which combined those who answered strongly support and somewhat support) or oppose (which combined those who answered neutral, somewhat oppose and strongly oppose). Including neutral responses with opposed ensures that our results are conservative and reduces misinterpretation that findings may be biased. Missing values were also recorded but excluded from the analysis.

### Independent (explanatory) variables

Socio-demographic covariates were chosen based on prior research [20,21]. The academic team used their reasoning to determine which covariates were of most importance to include in the survey. This was intended to reduce interview burden. Independent variables include: sex (male or female), age (19–34 years, 35–54 years, or 55 years and older), education level (less than high school, completed high school, some post secondary, completed diploma or certificate program, or completed university), and geographic location of residence (one of the five regional Health Authorities: Fraser Health, Interior Health, Northern Health, Vancouver Costal Health, Vancouver Island Health, determined by the first 3 characters of their postal code). Categorization by Health Authority region was considered appropriate in this analysis as public health, including harm reduction, is part of the Health Authorities’ mandate in BC.

### Statistical analysis

We aimed for a sample size sufficient to ensure 95% confidence intervals (CI) would be no wider than 5% in each Health Authority, which was calculated based on the assumption that support for harm reduction in each would be approximately 60%. This support estimate was based on previously reported public polls in regions of Canada after review of the literature [20-22]. We calculated the sample size in the Fraser Health region first (the Health Authority with the greatest population) and rounded up to the nearest hundred (n = 400). We chose to sample an equal number of participants in each of the five regional Health Authorities to obtain 2000 complete surveys. Thus, in comparison to the Fraser Health Authority, other health regions were oversampled. To better represent the provincial perspective, we applied a weighting variable in the analysis to allow the responses to be adjusted for the age-sex population structure in BC. For example, the weighting variable calculated for males aged 19–34 in Fraser Health was determined by dividing the proportion of males age 19–34 in Fraser Health obtained from Canadian census data by the proportion of males aged 19–34 in Fraser Health from our survey data. Population estimates were obtained from the Population Extrapolation for Organizational Planning with Less Error (P.E.O.P.L.E 36) [23].

A map depicting level of support by Health Authority was created using a geographic information system (ArcGIS® v10.0, Redlands, CA). Bivariate and multivariate analysis was conducted for each of the four outcomes of interest (SPSS® v.14.0, Armock, NY).

The chi square (χ^2^) test for bivariate analysis was used to investigate the association between support for each outcome of interest and each independent variable (predictor). A bivariate analysis was chosen to compare respondents who support the outcome of interest with those who do not support the outcome of interest, in order to determine characteristics of those less likely to support harm reduction strategies and services. Multivariate logistic regression was used to determine factors associated with support for each outcome of interest while adjusting for sex, age, education level, and health authority of residence. This model was chosen to adjust for these potential confounders and to measure the effect of the covariates which were hypothesized to play a role in support for harm reduction. Independent variables were retained in the multivariate model if the bivariate test indicated a potential association with the outcome (p ≤ 0.20). All independent variables met this criterion. The referent group for residence used in the multivariate analysis was Vancouver Coastal Health because this Health Authority region was the first to implement needle distribution programs, a supervised injection facility, and heroin assisted therapy trials.

Odds ratios were calculated to estimate the change in likelihood of support between independent variable categories (p = 0.05). Missing responses were excluded from analysis. ROC curves were examined to assess model goodness-of-fit.

## Results

A total of 2000 surveys were completed out of 8107 answered calls to eligible respondents: a 24.7% response rate. Of the surveys with complete demographic information (n = 2000), 50% of the sample was female, 28% were aged 19–34 years, 37% 35–54 years, and 35% were 55 years and older. Seven percent of respondents had less than high school graduation, and 30% had completed a university degree. Of those who met the analysis criteria, 1393 (76%) reported supporting harm reduction (n = 1834); 1396 (72%) supported needle distribution (n = 1929), 982 (65%) supported needle distribution in one’s community (n = 1507), and 975 (52%) supported distribution of safer inhalation equipment (n = 1880). Participant’s responses stratified by the 5 item Likert scale are displayed in Table 1.

**Table 1:** Participant responses to the outcome variables of interest (n = 2000)

**Outcome variables of interest**	**Level of support**				
	**Strongly support**	**Somewhat support**	**Neutral**	**Somewhat oppose**	**Strongly oppose**
	**n (%)**	**n (%)**	**n (%)**	**n (%)**	**n (%)**
General harm reduction	828 (45%)	565 (31%)	83 (5%)	166 (9%)	192 (10%)
*n = 1834*					
*Missing = 166*					
Needle distribution services	857 (44%)	539 (28%)	73 (4%)	159 (8%)	301 (16%)
*n = 1929*					
*Missing = 71*					
Needle distribution in one’s community	526 (35%)	456 (30%)	57 (4%)	156 (10%)	311 (21%)
*n = 1507*					
*Missing = 493*					
Safer inhalation equipment distribution	417 (22%)	558 (30%)	60 (3%)	272 (15%)	573 (30%)
*n = 1880*					
*Missing = 120*					

**Figure 1:**
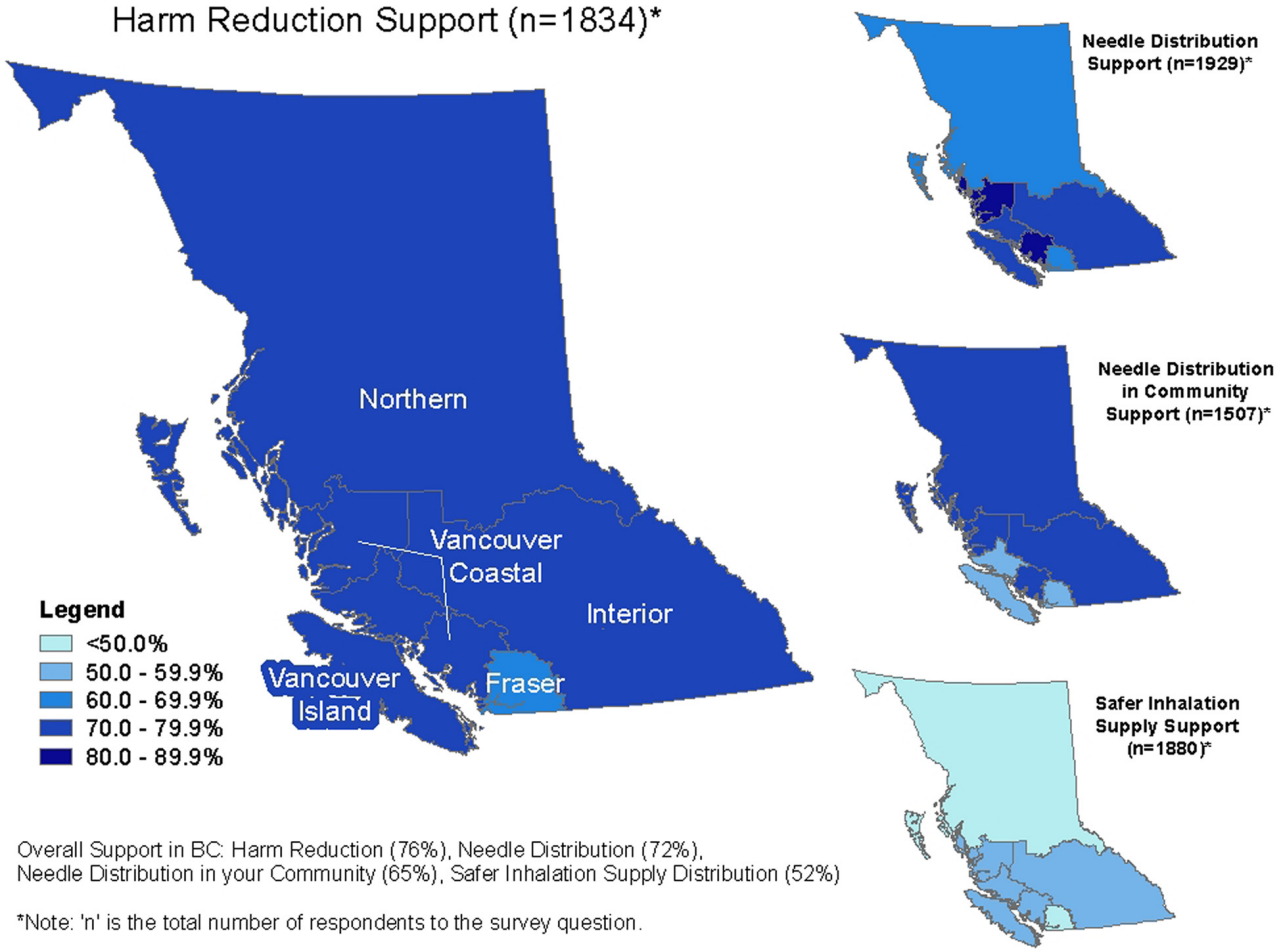
Maps of British Columbia describing the level of support for each of the outcomes of interest by Health Authority.

Figure 1 shows four BC maps to highlight the level of support by Health Authority for each of the outcomes of interest. Overall, the level of support across the Health Authorities was in a similar range although Fraser Health Authority’s level of support remained consistently lower than the others for each of the outcomes of interest. The figure shows that respondents supported harm reduction most, whereas the lowest level of support was for distribution of safer inhalation equipment. Only the analysis for the outcome ‘harm reduction’ is shown in detail in this manuscript.

The bivariate analysis (Table 2) shows that support for harm reduction is significantly associated with sex, age, education level, and health authority. Females, younger respondents, and those with a higher level of education were most supportive. The bivariate analyses for the other three outcomes of interest are not shown but the independent variables associated with support were found to be the same.

**Table 2:** Bivariate analysis of the association between predictors and support for harm reduction programs in British Columbia (n = 1834)

**Variable**	**Totals (Freq%)**	**Harm reduction programs**		
		**Support**	**Oppose**	p-value^*^
		***(n = 1393)***	***(n = 441)***	
**Sex***n (%)*				*0.001*
Female	981 (53.5)	776 (79.1%)	205 (20.9%)	
Male	853 (46.5)	617 (72.3%)	236 (27.7%)	
**Age**				*0.023*
19 – 34	335 (18.5)	269 (80.3%)	66 (19.7%)	
35 – 54	851 (46.4)	653 (76.7%)	198 (23.3%)	
55+	648 (35.3)	471 (72.7%)	177 (27.3%)	
**Education***n* (%)				*<0.001*
< High school graduation	138 (7.5)	95 (68.8%)	43 (31.2%)	
= High school	320 (17.4)	223 (69.7%)	97 (30.3%)	
= Some post secondary	321 (509)	252 (78.5%)	69 (21.5%)	
= Certificate/ diploma program	546 (39.8)	379 (69.4%)	130 (23.8%)	
= University graduate	546 (29.8)	444 (81.3%)	102 (18.7%)	
**Health authority***n* (%)				*0.009*
VCH	372 (20.3)	294 (79.0%)	78 (21.0%)	
IH	364 (19.8)	279 (76.6%)	85 (23.4%)	
FH	365 (19.9)	251 (68.8%)	114 (31.2%)	
VIHA	368 (20.1)	287 (78.0%)	81 (22.0%)	
NH	365 (19.9)	282 (77.3%)	83 (22.7%)	

*p-values from conventional χ^2^-tests for association between categorical variables.

In this summary, support includes ‘support’ and ‘strongly support’ responses and oppose includes ‘neutral’, ‘oppose’ and ‘strongly oppose’ responses.

The multivariate association between support for harm reduction and the independent variables is presented in Table 3. The odds of supporting harm reduction was 26% lower for males in comparison to females, after adjusting for age, education level, and Health Authority (AOR = 0.74, 95% Confidence Interval (CI) 0.59, 0.92). After adjusting for all independent variables, when compared to the reference group 19–34 years of age, participants 55 years and older were 37% less likely to support harm reduction (AOR = 0.63, 95% CI 0.48, 0.83). Compared to the referent group, university graduate, participants who had less than or equal to high school graduation were less likely to support harm reduction after adjusting for potential confounders (AOR = 0.51, 95% CI 0.33, 0.79; AOR = 0.70, 95% CI 0.51, 0.97, respectively), as were participants who completed a post secondary certificate or diploma program (AOR = 0.69, 95% CI 0.51, 0.92). Furthermore, participants residing in Fraser Health Authority were 41% less likely to support harm reduction in comparison to participants residing in Vancouver Coastal Health Authority, after adjusting for potential confounders. Again, the analyses for the other outcomes are not shown here, in which trends were found to be similar.

**Table 3:** Logistic regression models of predictors associated with support for harm reduction programs in British Columbia (n = 1834)

**Variables**	**Unadjusted**		**p-value**^**1**^	**Adjusted**		**p-value**^**2**^
	**OR**	**95% CI**		**OR**	**95% CI**	
**Sex**						
Female (referent) vs. Male	0.76	0.61, 0.94	*0.010*	0.74	0.59, 0.92	*0.006*
**Age**						
19 – 34 (referent)						
35 – 54	0.90	0.69, 1.20	0.481	0.89	0.67, 1.18	0.412
55+	0.64	0.49, 0.83	*<0.001*	0.63	0.48, 0.83	*0.001*
**Education**						
= University graduate (referent)						
< High school graduation	0.49	0.32, 0.76	*0.001*	0.51	0.33, 0.79	*0.002*
= High school	0.46	0.46, 0.86	*0.004*	0.70	0.51, 0.97	*0.030*
= Some post secondary	0.60	0.60, 1.16	0.271	0.88	0.63, 1.23	0.443
= Certificate/ diploma program	0.52	0.52, 0.93	*0.014*	0.69	0.51, 0.92	*0.012*
**Health authority***n* (%)						
VCH (referent)						
IH	0.91	0.64, 1.29	0.597	0.99	0.69, 1.41	0.934
FH	0.57	0.43, 0.75	*<0.001*	0.59	0.44, 0.78	*<0.001*
VIH	1.02	0.72, 1.45	0.914	1.12	0.78, 1.60	0.545
NH	0.92	0.56, 1.53	0.757	1.02	0.61, 1.69	0.948

^1^p-values from single d.f., Wald χ^2^-tests for each parameter in single predictor logistic regression models.

^2^p-values from single d.f. Wald χ^2^-tests for each parameter in a multiple predictor logistic regression model including Sex, Age, Education, and Health Authority.

## Discussion

Our findings show an overall high level of support for harm reduction strategies and services among participants in our survey. However, when respondents were asked about support for needle distribution programs in one’s community or for safer inhalation equipment, overall level of support was lower than for more general harm reduction. The results indicate that females, younger individuals, those with higher levels of education, and those residing outside the Fraser Health Authority are more supportive of safer drug use initiatives.

These findings are consistent with other Canadian literature and opinion polls regarding harm reduction support. A 2007 Canadian national public opinion poll showed that those with greater income and education level were most supportive of harm reduction efforts in Canada [24]. A study from Ontario showed an association between education level and support for supervised injection facilities and heroin-assisted treatment [21]. It is possible that spending time in an educational environment exposes one to information and debate about problematic substance use and a better understanding of the framework for harm reduction [21]. The literature on health literacy also finds that individuals with lower levels of education and/or individuals not exposed to socio-scientific thinking may be less likely to critically review and understand the role of harm reduction programs [25-27]. Socio-scientific thinking involves analyzing stakeholder perspectives, ethical principles, and relevant scientific findings [25]. Furthermore, one of the major provincial newspapers in BC often negatively represents harm reduction. This is not surprising as the media tend to support dominant moral conceptions, of which harm reduction may not be perceived as one [28]. We have limited knowledge of how the general population uses and interprets media [29] but the negative articles of this newspaper could influence opinions formed by its readers [19,30]. Alternatively, this paper’s readership was found to have lower levels of education in comparison to another major newspaper in BC [19]; thus, the readers of this paper may tend to agree with viewpoints published in this paper. This is a relationship that warrants further exploration as it is not consistent with some policies in BC or the opinions of our participants.

Older persons were found to be less supportive of harm reduction strategies than younger persons. Harm reduction has been introduced relatively recently in BC with the first needle/syringe program opening in 1989. The ‘Just Say No’ campaign in the 1980s and the ‘Reefer Madness’ campaign before that may have influenced popular understanding at the time of what is effective in reducing drug use and drug-related harms [18,31-33]. This may have led to the adoption of these ideologies in older generations who now find it difficult to understand the role of harm reduction, along with prevention, treatment and law enforcement, to address problematic substance use. Efforts should be made to engage older individuals in discussing and learning about the public health issues surrounding problematic substance use, safer drug use practices, and effective harm reduction strategies. Harm reduction is ultimately a cost-saving measure that connects people who use drugs to medical care and refers them to addiction services. Contrary to concerns expressed by some segments of the public, evidence shows harm reduction does not encourage drug use and may decrease drug use [34,35]. Harm reduction is most effective in reducing the transmission of blood-borne pathogens if it is widely available in low barrier settings [36-38]. Lastly, harm reduction services are a human right [39].

We found less support for harm reduction in the Fraser Health Authority region, in which a bylaw prohibiting harm reduction services is in place in several municipalities [10,39]. Despite this, the majority (69%) of respondents from the Fraser Health Authority region reported support for harm reduction. This brings into question what proportion of Fraser Health Authority residents truly support the bylaws and whether these were enacted as a result of vocal minorities. Another municipality that closed a needle distribution site was found to have 78% support for harm reduction in their Health Authority. MacNeil and Pauly [12] noted that there was a strong local community presence that led to the closure of this 20 year old distribution site on Vancouver Island. In these two situations policy makers may have been swayed by a vocal minority and/or may have blocked harm reduction efforts for other reasons, but the evidence suggests there may be a high level of public support for harm reduction efforts in these Health Authorities. Looking deeper into community support for harm reduction programs may help address this anomaly.

Of the harm reduction strategies discussed in our survey, the research team noted that the greatest number of missing responses was for the question: “Do you support needle distribution in your community?” (See Table 1). This was in contrast to the previous question which asked about general support of needle distribution programs. Respondents may have experienced cognitive dissonance when asked this question, as they may support harm reduction and needle distribution in theory, but were not comfortable to admit that they preferred this service not be conducted close to their homes. This NIMBY attitude has also been documented in conjunction with other ‘less desirable’ services (e.g. jails and homeless shelters) in city planning [16,39]. However, our findings show the majority of respondents supported needle distribution programming in their community. Furthermore, members of the community may be more likely to change their attitude and say ‘yes-in-my-backyard’ (YIMBY) when community concerns have been addressed [3,39].

Addressing community concerns is particularly important as one Canadian study found that the anticipated community resistance to harm reduction was the biggest barrier for service providers to implementing harm reduction programming [40]. This anticipated concern has been quantified in our study as overall support for community needle distribution was lower than general needle distribution. This is not to suggest that program implementation should be halted due to perceived lack of community support, but instead we encourage service providers to take the time, care, and effort with their community in order to build sustainable harm reduction programming. As well, Bernstein and Bennet [39] suggest the harm reduction movement should engage with municipalities in the city’s planning of health and social services.

Of the harm reduction strategies surveyed, safer inhalation equipment distribution was least supported (See Figure 1). This is the newest of the harm reduction strategies and has received negative press. The public may be less informed about the harms associated with crack cocaine inhalation and the health impacts of makeshift equipment which include: throat and mouth damage, burn on lips, and hepatitis C transmission [35,41]. Many respondents may not have been aware that hepatitis C virus has been found on crack pipes, which if shared could be a route of hepatitis C transmission [41]. It should also be noted that our survey was administered during a negative media blitz regarding Vancouver Coastal Health’s crack kit pilot project [42,43] that had begun the weekend prior to the administration of our survey.

Another interesting finding is the polarization of the participant’s responses. Very few participants answered ‘neutral’, and for the most part the majority of responses were stated as ‘strongly support’ or ‘strongly oppose’. This may suggest that respondents have already formed strong opinions. Thus, it may take time to shift the opinion of those who strongly oppose harm reduction as they may have deep rooted feelings about safer drug use practices.

There are a number of limitations to this study. Firstly, BC residents without a landline telephone number were excluded. The response rate was 24.7%. Overall, this is a low response rate but it is consistent with or better than other public polls. The average response rate of 12–15 minute telephone surveys is 24%, and 12% for random-digit dialing sampling [44]. A high profile public surveying company also found that telephone polling refusal rates can reach 90% [45]. Current reports show the public is overburdened with requests to participate in telephone surveys and periodically even the most robust polling methods may lead to false outcomes [45]. There are no other methods of understanding public opinion better than polling [45], yet the authors of this study ask the audience to use caution when interpreting our results. We encourage more research in this field as governments may be less likely to undertake public opinion polls in case the results contradict current policy. Public polls have an important place in society and may promote policy changes [45].

In this study, refusals to participate may have been due to negative attitudes towards harm reduction at survey introduction. Social desirability bias may have also played a role in responses. Providing a description about the harm reduction strategy first may have led the respondent to change their views; however, the response would be more informed. This telephone survey provided an estimate of the public’s opinion and the large sample size taken by random digit dialing attempted to reduce inherent biases as much as possible. We were able to compare the five Health Authorities by using a weighted sample; however, our sample size was not sufficient to explore geographic areas smaller than the regional health authority level. Furthermore, we were unable to select participants based on the population’s education level. Previously conducted telephone surveys in Canada found that those with higher level of education had been overrepresented [21]. To examine the role of education in our study we included education level in the multivariable model. Lastly, to ensure acceptability of survey length by participants, the research team limited the number of independent variables asked. Despite this, the analysis provides insights into the attitudes of safer drug use practices in BC. Readers should also be aware that this analysis took a conservative approach by including ‘neutral’ responses with ‘opposed’.

## Conclusion

There was a high level of support for harm reduction strategies and services among participants in this public opinion poll in British Columbia, Canada. The findings are encouraging for those working in harm reduction who may face vocal opposition to their work as these opposing views may not reflect the general public’s attitudes. Policies limiting harm reduction services may exist even in the presence of public support for harm reduction activities. This documented support for harm reduction among participants in our study, along with the body of evidence that harm reduction strategies are effective in preventing disease and improve quality of life, should be reflected in policies and be used to garner support for program implementation in BC and internationally. Our findings may encourage researchers in other areas of the world to conduct similar studies in order to better understand the general public’s attitudes towards safer drug use practices in their region, in addition to developing strategies to increase public support and political will.

## Abbreviations

BC: British Columbia; NIMBY: Not-in-my-back-yard; US: United States; YIMBY: Yes-in-my-back-yard.

## Competing interests

The authors declare that they have no competing interests.

## Authors’ contributions

The specific contribution of each author is as follows: JC, MK, and JB were responsible for study design; DT and JC were responsible for collection of the literature and first draft. DT, MK, and JB were responsible for analysis. All authors provided critical comments on the first draft of the manuscript. All authors read and approved the final manuscript.

## Authors’ information

All authors are affiliated with the Communicable Disease Prevention and Control Services (CDPACS) at the British Columbia Centre for Disease Control located at 655 West 12th Avenue, Vancouver, BC V5Z 4R4, Canada. CDPACS is responsible for the British Columbia’s Provincial Harm Reduction Program. Information about the Harm Reduction Program is available through the following websites: http://www.bccdc.ca/prevention/HarmReduction/default.htm and http://www.towardtheheart.com or through communication with the corresponding author.
